# Redirecting Specificity of T cells Using the *Sleeping Beauty* System to Express Chimeric Antigen Receptors by Mix-and-Matching of V_L_ and V_H_ Domains Targeting CD123^+^ Tumors

**DOI:** 10.1371/journal.pone.0159477

**Published:** 2016-08-22

**Authors:** Radhika Thokala, Simon Olivares, Tiejuan Mi, Sourindra Maiti, Drew Deniger, Helen Huls, Hiroki Torikai, Harjeet Singh, Richard E. Champlin, Tamara Laskowski, George McNamara, Laurence J. N. Cooper

**Affiliations:** 1 Division of Pediatrics, The University of Texas MD Anderson Cancer Center, Houston, Texas, United States of America; 2 The University of Texas Graduate School of Biomedical Sciences at Houston, Houston, Texas, United States of America; 3 Stem Cell Transplantation and Cellular Therapy, The University of Texas MD Anderson Cancer Center, Houston, Texas, United States of America; 4 Ziopharm Oncology Inc., Boston, Massachusetts, United States of America; 5 Intrexon Corporation, Germantown, Maryland, United States of America; 6 Surgery Branch, Center for Cancer Research, National Cancer Institute, National Institutes of Health, Bethesda, Maryland, United States of America; Jackson Laboratory, UNITED STATES

## Abstract

Adoptive immunotherapy infusing T cells with engineered specificity for CD19 expressed on B- cell malignancies is generating enthusiasm to extend this approach to other hematological malignancies, such as acute myelogenous leukemia (AML). CD123, or interleukin 3 receptor alpha, is overexpressed on most AML and some lymphoid malignancies, such as acute lymphocytic leukemia (ALL), and has been an effective target for T cells expressing chimeric antigen receptors (CARs). The prototypical CAR encodes a V_H_ and V_L_ from one monoclonal antibody (mAb), coupled to a transmembrane domain and one or more cytoplasmic signaling domains. Previous studies showed that treatment of an experimental AML model with CD123-specific CAR T cells was therapeutic, but at the cost of impaired myelopoiesis, highlighting the need for systems to define the antigen threshold for CAR recognition. Here, we show that CARs can be engineered using V_H_ and V_L_ chains derived from different CD123-specific mAbs to generate a panel of CAR^+^ T cells. While all CARs exhibited specificity to CD123, one V_H_ and V_L_ combination had reduced lysis of normal hematopoietic stem cells. This CAR’s *in vivo* anti-tumor activity was similar whether signaling occurred via chimeric CD28 or CD137, prolonging survival in both AML and ALL models. Co-expression of inducible caspase 9 eliminated CAR^+^ T cells. These data help support the use of CD123-specific CARs for treatment of CD123^+^ hematologic malignancies.

## Introduction

Immunotherapy holds great promise for improving outcomes for some of the worst cancers, including acute myelogenous leukemia (AML). Tremendous advances have been seen in recent years from several applications of immune-based treatment [1–3], especially those that exploit the precise antigen recognition of monoclonal antibodies (mAbs). An especially promising development has been the creation of chimeric antigen receptors (CAR) for T cells [4], utilizing single chain polypeptides encoding the V_H_ and V_L_ domains (scFv) of a mAb, coupled with a transmembrane domain and the CD3ζchain. Second generation CARs include the signaling domain of either CD28 [5, 6] or CD137 [7–9] to provide “signal 2,” which is essential for improved activation and function, as well as for prolonged T cell survival. The use of CAR^+^ T cells whose antigen recognition has been redirected to specific tumor associated antigens (TAA) for adoptive immunotherapy has already provided remarkable success in early phase clinical trials [10–12], though several important questions remain regarding optimal CAR design and choice of TAA for an increasing range of malignancies. Some of these key questions include how to tune the sensitivity of CAR^+^ T cells to recognize the increased levels of TAA on tumor cells while avoiding the toxicities that arise from recognition of normal cells [13], and which costimulatory signal provides the best phenotype and persistence for CAR^+^ T cells.

Establishment of long term memory and survival is vital for improving anti-tumor efficacy of CAR^+^ T cells in the clinical setting. Terminally differentiated effector memory (T_EM_) T cells lose their capacity to expand and persist after adoptive transfer [14–16]. Conversely, less differentiated central memory (T_CM_) T cells can further expand, differentiate, or self-renew, providing superior clinical response [14]. To date, adoptively transferred CAR^+^ T cells have demonstrated significant antitumor activity but limited *in vivo* expansion in clinical applications [17–19]. Though interleukin-2 (IL-2) is routinely used for T cell expansion, recent reports suggest that other common gamma chain cytokines, such as IL-15 and IL-21, suppress the differentiation of naïve T cells into effector T cells and may be more useful for adoptive therapy purposes [20]. For effective adoptive immunotherapy, it is ideal to infuse cells in an early state of maturation, as these cells retain the best persistence potential and anti-tumor efficacy [15, 21].

An additional challenge in developing CAR^+^ T cells for immunotherapy is toxicity management, especially those toxicities related to excess activation of infused cells [22–24] or targeting of TAA expressed on normal tissues [25]. These concerns have led some to suggest that genetically modified T cells should include an inducible “suicide switch” or other mechanism to terminate responses should toxicity become excessive [23].

Some of the best early responses from CAR T cell therapy have been in treating lymphoid malignancies, especially by targeting CD19 expression [6, 12, 26–31]. Less is known about the utility of CAR therapy for AML, for which conventional therapy provides only a 30–50% long-term remission rate and an adverse outcome in the majority of patients diagnosed [32–34]. Relapse in AML, similar to ALL, is the result of residual, often subclinical, disease consisting of leukemic stem cells (LSCs) remaining after maximal conventional therapy. LSCs typically are resistant to both chemotherapy and radiation, highlighting the need for alternative approaches to improve outcomes. Immunotherapy directed against AML TAAs offers such an approach, though responses of AML to monoclonal antibodies have been disappointing, and antibody conjugate therapies have had difficulties with excess toxicities and provided modest improvements in outcome at best [35, 36]. CAR T cell therapy for AML would offer exciting new possibilities for treating poor- prognosis AML.

One potential TAA to target for AML therapy is CD123, the IL-3 receptor α subunit (IL3Rα). CD123 is overexpressed by up to 95% of leukemic blasts, including LSCs in AML and a majority of B cell acute lymphoblastic leukemia (B-ALL) blasts, but is low or absent on normal hematopoietic stem cells (HSC) or cells outside the hematopoietic lineage [37–41]. Phase 1 clinical trials targeting CD123 in AML using neutralizing mAbs and cytotoxic proteins fused to IL-3 cytokine or IL3Rα showed limited therapeutic efficiency [42–44], but establishing CD123-specific CAR T cell therapy has the potential to target AML LSCs and improve survival [25]. Additionally, CD123 may be a useful target for relapsed ALL, since some patients treated with CD19-specific CAR T cells relapsed with CD19^neg^ CD123^+^ disease [11, 45].

The optimal design for a CD123-specific CAR is unknown, as is the optimal signaling domain for delivery of signal 2 in this context. The main goal of this study is to redirect T-cell specificity toward CD123 via CAR to target AML and to generate preclinical data in support of an adoptive immunotherapy trial using this novel CAR for both AML and B-ALL. We describe CD123-specific CARs using chimeric scFvs derived by mixing and matching V_L_ and V_H_ chains of different mAbs specific for CD123, testing the activity of T cells genetically modified via the S*leeping beauty* (SB) system [46–48] against AML and ALL cells as well as normal bone marrow (BM)-derived cells. We also describe the comparative evaluation of CD123-specific CARs with CD28 or CD137 co-stimulatory domains. Finally, we tested the efficacy of CD123-specific CAR^+^ T cells against *in vivo* models of both AML and ALL.

## Materials and Methods

### Generation of CD123 specific CARs with scFvs derived from two monoclonal antibodies

**Fig 1:**
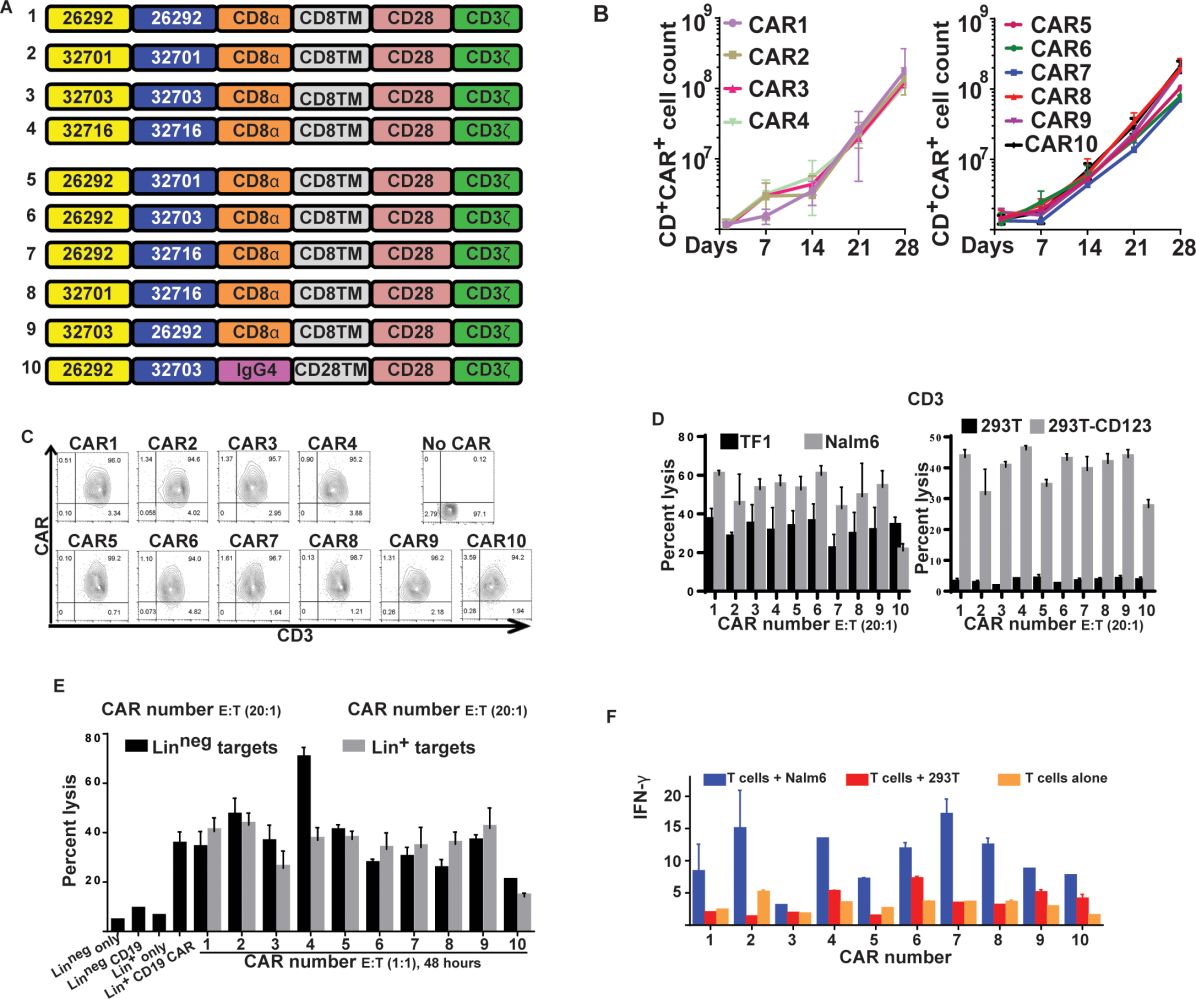
Production and testing of CD123-specific CARs with chimeric scFvs. **(A)** Schematic diagrams of conventional and chimeric scFv specific for CD123. CARs 1 to 4: CD123-specific CARs generated by fusing V_L_ and V_H_ chains of mAbs specific to CD123. CARs 5–9: Chimeric scFvs created by mix-and-matching V_L_ and V_H_ chains. The scFvs of CARs 1–9 were fused to the signaling domains of CD28 and CD3ζ via CD8α hinge and TM domains. CAR-10 was derived by fusing the chimeric scFv from CAR 6 to the CD3ζ and CD28 endo-domains via the IgG4 hinge and CD28 TM domains. **(B)** Expansion kinetics of CARs 1–4 (left) and CARs 5–10 (right) over a period of 28 days from day 1 following electroporation of SB CAR plasmids. Data are pooled from 3 donors; graph displays mean ± SEM **(C)** CAR expression on Day 21 after electroporation. CAR expression was detected by CD123 recombinant protein fused to Fc followed by serial staining with fluorescence-labeled anti-Fc and anti-CD3 antibodies. **(D)** *in vitro* lysis of CD123^+^ target cells Nalm 6, TF1, 293T-parental cells, CD123-transfected 293T cells, and 123^neg^ by CAR^+^ T cells. Histograms represent the mean ± SEM, n = 3. **(E)** CAR^+^ T cell killing of BM-derived target cells. Mononuclear cells were isolated from normal human bone marrow samples and sorted for expression of lineage markers into lineage-positive (Lin^+^) and lineage-negative (Lin^neg^) groups. The latter presumable reflects the HSC pool. The BM-derived cells were then labeled with PKH-26 and incubated with CAR^+^ T cells for 2 days before vitality was assessed by flow cytometry. The percent lysis compared to controls is shown. Histograms represent the mean ± SEM of 3 replicates. (F) Interferon-γ release by CAR^+^ T cells after exposure to CD123. Day 28 CD123-specific CAR^+^ T cells were incubated for 24 hours with Nalm-6 cells (CD123^+^), 293T cells (CD123^neg^), or alone, then the supernatant tested for cytokine expression using Biolegend plex Th1 cytokine capture beads, measured by flow cytometry. Results for IFN-γ are shown; other cytokines were not detectible over background. Histograms represent mean ± SEM for 2 replicates from 2 different experiments.

To generate CARs specific for CD123, we used scFvs from four monoclonal antibodies specific for CD123 (clones 26292, 32701, 32703 and 32716)[49], which were then fused in frame to the human CD8α spacer and transmembrane domain, then the CD3ζ and CD28 endodomains, to generate CARs 1–4 (**Fig 1A**). Of several possible chimeric scFvs that can be made, we chose five for further testing. These five mix-and-match scFvs were spliced into the existing anti-CD123 CAR construct described above to generate CARs 5–9 (**Fig 1A**). CAR 10 has the same scFv as CAR 6, but uses the IgG4 spacer and CD28 TM. CAR constructs were custom-synthesized and cloned into SB system constructs, as described previously for CD19 CARs [48].

### Construction of iCaspase 9^+^ CARs in SB transposons

For experiments testing the relative contributions of CD28 vs. CD137 signaling as the costimulation signal for CAR T cells, we chose the CAR10 scFv described above (**Fig 1A**), since we have previously engineered CAR constructs using these costimulatory domains fused to the IgG4 transmembrane domain. On the 5’ side of the resulting CAR sequence, there is an in- frame inducible caspase 9 sequence (iCasp9) [50, 51], followed by a Furin element and F2A peptide sequence, which together make an auto-cleavage site within the protein, resulting in two mature proteins from the single polypeptide sequence [52]. The iCasp9 element creates a chemically inducible suicide switch in CAR^+^ cells. This entire construct (iCasp9-Furin-F2A- CAR10) was inserted into the SB transposon vector, as described previously [47, 53, 54]. The vector maps for these constructs are provided in **S1 Fig.** The CAR constructs were custom synthesized and codon optimized by Geneart, (Invitrogen, Grand Island, NY) and cloned into SB vectors. The sequence for both plasmids was verified by Sanger sequencing (DNA Sequencing Core, MD Anderson).

### Primary cells and cell lines

The TF1 cell line was obtained from the European Collection of Authenticated Cell Cultures (ECACC). Molm13, MV411, and OCI-AML3 were kind gifts from Dr. Dean A. Lee (MD Anderson). EL4 cells were obtained from American Type Culture Collection (ATCC). RCH-ACV and Kasumi-2 were kind gifts from Jeffrey Tyner (Oregon Health Sciences University). OCI-Ly19 was a kind gift from Dr. Richard Eric Davis (MD Anderson). K562-derived Activating and Propagating Cells (AaPC) were obtained from Dr. Carl H. June (University of Pennsylvania) and further modified with membrane bound (m)-IL15, Receptor Tyrosine Kinase-Like Orphan Receptor-1 (ROR1) and the TAA CD123 (see below). The Nalm-6 cell line was obtained from Deutsche Sammlung von Mikroorganismen und Zellkulturen (DSMZ). Peripheral blood mononuclear cells (PBMC) for T cell transfections were obtained from healthy donors after informed consent and isolated by density gradient centrifugation using Ficoll-Paque^™^ PLUS (GE Healthcare). All cell lines were maintained in complete RPMI media, 10% FBS and 1X Glutamax-100. STR DNA fingerprinting was done to confirm the identity of all cell lines at MD Anderson’s Cancer Center Support Grant (CCSG) supported facility “Characterized Cell Line Core.”

### Generation of CD123^+^ Clone1-APC, EL4 and 293T cells

To generate AaPC to support expansion of CD123-specific CAR^+^ T cells, we modified K562-based AaPC originally obtained from Carl June (Clone 9) which express CD19, CD64, CD86, and CD137L [55], to express an IL15/IL15Rα fusion protein (**S2 Fig**), ROR1 (**S3 Fig**) and CD123 (**S4 Fig**) using SB gene transfer according to our published methods [48]. This new AaPC line we termed Clone1-CD123. The same process was used to create EL4 and 293T cells expressing CD123. CD123^+^ cells were selected with hygromycin.

### Electroporation and propagation of CAR^+^ T cells

CAR^+^ T cells were produced from PBMC as described [54, 56, 57]. Following electroporation with transposon- and transposase-containing plasmids, 20 million PBMC were cultured overnight, then stimulated with γ-irradiated (100 Gy) Clone 1-CD123 at a 1:2 ratio of T cells to AaPCs, supplemented with 50 units/ml recombinant human IL-2 (Prometheus Laboratories) and 30 ng/ml recombinant human IL-21 (Pepro Tech). AaPCs were added every 7 days, and IL-2 and IL-21 were added Monday, Wednesday and Friday of each 7 day T cell expansion cycle. T cell cultures were phenotyped by flow cytometry weekly to monitor CAR expression. Outgrowth of NK cells (CD3^neg^CD56^+^ population) typically was observed 10 to 14 days after electroporation. If the percentage of NK cells exceeded 10%, NK cells were depleted with CD56 beads (cat.no.130-050-401, Miltenyi Biotech) according to the manufacturer’s instructions. As a positive control, 5 × 10^6^ PBMC were mock-transfected without CAR plasmid or transposase and co-cultured on γ-irradiated (100 Gy) anti-CD3 (OKT3) loaded K562-AaPC Clone #1 at a ratio of 1:1 in a 7-day stimulation cycle along with IL-2 and IL-21 as described for CAR T cells above.

### Real time PCR to determine integrated CAR copy number

The number of integrated copies of the CD123-specific CAR transgene was determined from genomic DNA as described [58]. Genomic DNA from a genetically modified Jurkat T-cell (clone#12) containing 1 copy of CAR per cell from the CoOpCD19RCD28/pSBSO DNA plasmid [59] was used as a positive control. No DNA (CAR^neg^) T cells were used as negative controls. Results were analyzed using GraphPad Prism software.

### Immunophenotype of CAR^+^ T cells

The immunophenotype of SB gene-modified T cells was assessed by flow cytometry using appropriate antibodies (**S5 Fig**) and isotype controls as described [60]. For intracellular staining of the FLAG domain in the iCasp9 construct, cells were fixed and permeabilized for 20 minutes at 4°C with BD Cytofix/Cytoperm (BD Biosciences, San Diego, CA) followed by staining with appropriate antibodies. All samples were acquired on a FACSCalibur (BD Bioscience) and analyzed with FlowJo software (version 7.6.3).

### Multiplex Gene Expression Analysis of CAR T cells

After 35 days of co-culture with AaPC, 10^5^ CAR^+^ T cells were lysed in 17 μl of RLT buffer (Qiagen) and frozen at -80°C. Cell lysates were thawed and analyzed immediately using the nCounter analysis system (NanoString Technologies, Seattle, WA) with the “lymphocyte codeset array” as described [61]. Data was normalized to known amounts of added positive control RNA and housekeeping genes (*ACTB*, *G6PD*, *OAZ1*, *POLR1B*, *POLR2A*, *RPL27*, *Rps13*, and *TBP*), where 2 normalization factors were calculated and applied to the raw counts. Each normalization factor was calculated from the average of sums for all samples divided by the sum of counts for an individual sample. Total counts for LCA genes described in CD123-specific CAR^+^ T cells were directly reported as normalized mRNA counts.

### iCaspase 9 functional assay

CAR^+^ T cells were seeded in 24-well plates at a concentration of 10^6^ cells/well and treated with and without 1 μM of chemical inducer of dimerization (CID) (AP20187; Clontech). Untreated CAR^+^ T cells were used as controls. Cells were harvested after 24 hours and surface stained with Fc-PE to detect the IgG4 hinge of the CAR T cells, followed by annexin-V and 7-amino- actinomycin D (7-AAD) staining according to the manufacturer's instructions (BD Pharmingen). Data were acquired by FACSCaliber (BD Bioscience) and analyzed by FlowJo software (version 7.6.3).

### Chromium release assay

The cytolytic efficacy of CAR^+^ T cells with target cell lines was evaluated using a 4-hour chromium release assay as described [48]. Data are reported as mean ± SD

### Flow cytometric killing assay

For T cell killing assays in AML primary samples, target cells were labeled with PKH-26 (Sigma, cat. no PKH26PCL) according to the manufacturer’s instructions and co-cultured with CAR^+^ T cells at an E:T ratio of 1:1 for 3 days without exogenous cytokines. For *in vitro* lysis of normal BM targets lineage^+^ and lineage^neg^ cells were isolated from BM mononuclear cells (All cells, cat. no. ABM024) using the Diamond CD34 isolation kit (Miltenyibiotec, cat. no.130-094-531) according to the manufacturer’s instructions. BM targets were labeled with PKH-26 and co-cultured with CAR T cells for 48 hours at an E:T ratio of 1:1. 7-AAD staining was used to exclude dead cells, and viable cells were PKH26^+^ and 7-AAD^neg^.

### Cytokine production by CAR^+^ T cells

Effector cells were incubated with target cells at T cell to target ratio of 1:1 for 24 hours. Cytokine production from CAR^+^ T cells in response to antigen was determined using LEGENDplex™ Multi-Analyte Flow Assay Kit (Biolegend, cat.no 790004) according to the manufacturer’s instructions, and analyzed by iQue Screener Plus (IntelliCyt Corporation, Albuquerque, NM).

### Mouse studies

The *in vivo* antitumor efficacy of CAR^+^ T cells was assessed in NOD/SCID/IL-2Rγ^-/-^ (NSG) mice transgenic for human IL-3, stem cell factor, and granulocyte macrophage colony-stimulating factor (GM-CSF) obtained from Jackson Laboratories. For bioluminescent xenograft models, the TF1 and RCH-ACV cell lines were genetically modified to express enhanced firefly luciferase (effLuc) (**S6 Fig and S7 Fig**) by transduction with a pLVU3G effLuc-T2A-mKateS158A lentivirus construct and sorting for uniform mKate expression as described [62, 63]. For the TF1 AML model, 12 NSG mice in each experiment were injected intravenously (i.v.) with 2.5 × 10^6^ TF1-effLuc cells, then divided into three groups of 4 mice each. On day 5, mice were injected with 10^7^ cells CD123-CD28 (group 2), or CD123-41BB CAR^+^ T cells (group 3) per mouse, or were given no cells (group 1). Tumor engraftment was confirmed by bioluminescent imaging (BLI) before T cell infusion. Additional T cell infusions were administered on days 11 and 20, and the tumor burden was assessed serially by BLI. The experiment was performed twice; one representative experiment is shown. For the RCH-ACV ALL model, 8 NSG mice were injected with 2.5 × 106 RCH-ACV-effLuc cells. Half were injected with 10^7^ CD123-CD28 CAR^+^ T cells on days 1, 7, 14 and 21. Both experimental and control mice received IL-2 (60,000 units/mouse) on days 1, 7, 14, and 21, followed by BLI to assess tumor burden. This experiment was performed twice; a representative experiment is shown.

### Ethics statement

All patient samples used for this study were obtained after written informed consent was obtained in accordance with protocols established and approved by the MD Anderson Internal Review Board (IRB). The samples were de-identified. Animals were handled in accordance with the strict guidelines established by the MD Anderson cancer center Institutional Animal Care and Use Committee (IACUC). The animal protocol was approved by IACUC. All efforts were made to minimize animal suffering and inhaled isoflurane was administered for anesthesia as required.

## Results

### CD123-specific CARs with chimeric scFvs

CD123 is a likely target for CAR-based immunotherapy for AML, but the optimal design for a CD123-specific CAR is not known. CARs utilizing the V_H_ and VL from the same mAb are known to function but the utility of CARs utilizing V_H_ and V_L_ chains from different mAbs is less clear. Using CARs designed from four CD123-specific mAbs (26292, 32701, 32703, and 32716, designated CARs 1–4 respectively), we created five CARs in which the V_H_ and V_L_ were derived from different mAbs (**CARs 5–9, Fig 1A**). An additional construct (**CAR 10, Fig 1A**) utilized the same scFv as CAR6, but with the IgG4 hinge and spacer region in place of CD8-derived sequences. All ten CARs were introduced into PBMCs using the *SB* system as described (54, 56, 57). Recurrent stimulations were performed every 7 days for 4 weeks. The cells expanded at similar rates **(Fig 1B)**, and by 21 days all cultured lymphocytes were ˃ 90% positive for CD3 and CAR **(Fig 1C)** and lysed CD123^+^ tumors **(Fig 1D)**. Lysis was antigen specific, since all CAR^+^ T cells lysed 293 T cells gene-modified with SB to express CD123 but not parental cells. Importantly, all CAR constructs showed some lytic activity against normal BM-derived cells, whether the BM cells expressed lineage markers or expressed no lineage markers, presumably representing the stem cell pool **(Fig 1E)**. CAR 10 had the lowest activity against normal BM-derived cells. Most CARs conferred the ability of T cells to release IFN- γ following co-culture with CD123^+^ targets (**Fig 1F**).

### Engineering CD123-specific CARs with CD28 or CD137 and iCaspase 9

**Fig 2:**
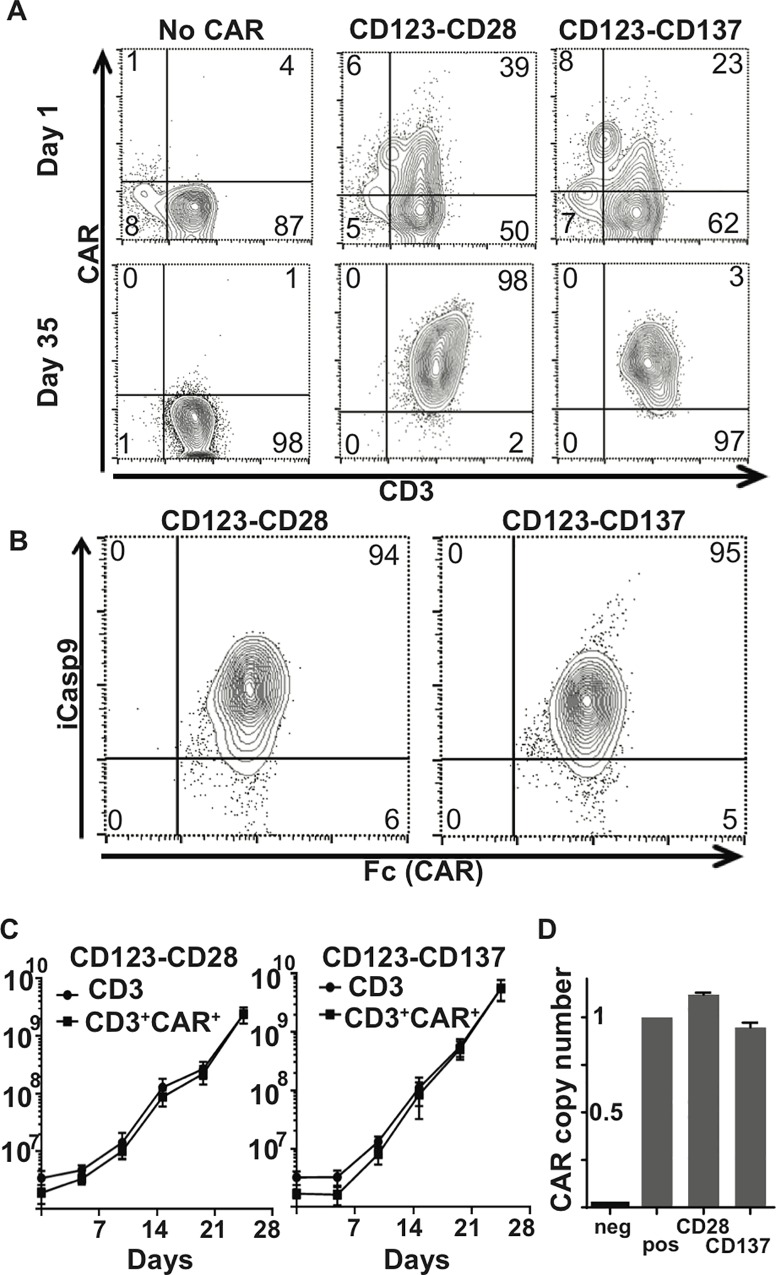
Production and testing of CD123 specific CAR^+^ T cells with CD28 vs. CD137 costimulatory domains. (**A**) CAR expression in CD123-CD28 (**middle**) and CD123-CD137 (**right**) CAR^+^ and CARneg T cells (**left**) on day 1and day 35 after electroporation and co-culture on AaPC, Clone 1-CD123. T cells were detected with fluorescence-labeled anti-CD3, and CAR expression with fluorescence-labeled Fc-specific antibody binding to the IgG4 hinge. (**B**) Expression of iCasp9 in CAR^+^ T cells. The same cells tested in (**A**) were examined by flow cytometry for surface expression of IgG4 hinge by Fc binding, and intracellularly for iCasp9 expression. (**C**) Expansion kinetics of CD123-CD28 (**left**) and CD123-CD137 CARs (**right**). Total CD3^+^ cells and CD3^+^CAR^+^ T cells expanded on AaPC Clone 1-CD123 over a period of 35 days. Graph shows the mean ± SEM of three donors. (**D**) CAR copy number was determined on day 28 using primers and probes specific for the CD28 transmembrane and IgG4 hinge regions. CAR^neg^ and CAR^+^ Jurkat cells were used as negative and positive controls respectively, Histograms represent the mean ± SEM, n = 3

Several pre-clinical and animal models have demonstrated that CAR^+^ T cells that include CD28 or CD137 co-stimulatory domains as a built-in source of signal 2 have improved persistence compared with those containing the CD3ζ signaling domain alone (5,6,8). However, the anti-tumor efficacy of one over the other costimulatory domain has not been investigated in depth. In addition, the utility of the inducible suicide switch iCasp9 has not been evaluated in this context. To address these questions, we engineered constructs in which the CAR10 CD123-specific second generation CAR was fused to either the CD28 (designated as CD123-CD28 CAR) or CD137 (designated as CD123-CD137 CAR) co-stimulatory domains, which were then inserted 3’ of the iCasp9 sequence [64] and a Furin-F2A autocleavage peptide linker [52], encoding a single polypeptide that results in two mature proteins. This construct was then inserted into the SB construct, as previously described [47, 53, 54] **(S1 Fig)**. PBMC from normal donors were co-electroporated with the CD123-CD28 or CD123-CD137 transposon and SB11 transposase plasmids and co-cultured with clone1-CD123 AaPC with IL-2 and IL-21 stimulation as described [54, 56, 57] for 5 weeks. By day 35, more than 95% of T cells expressed CD3 and CAR **(Fig 2A)** and iCasp9 **(Fig 2B).** Both CARs expanded at similar rates **(Fig 2C)**. Both constructs yielded an average integration of one copy of CAR transgene per cell. **(Fig 2D)**. Thus SB transposition of a CD123-specific CAR into PBMC was phenotypically similar with either CD28 or CD137 as the source of signal 2, and was not impaired by inclusion of iCasp9 in the construct.

### Immunophenotype and transcriptional profile of CD123 CAR^+^ T cells

**Fig 3:**
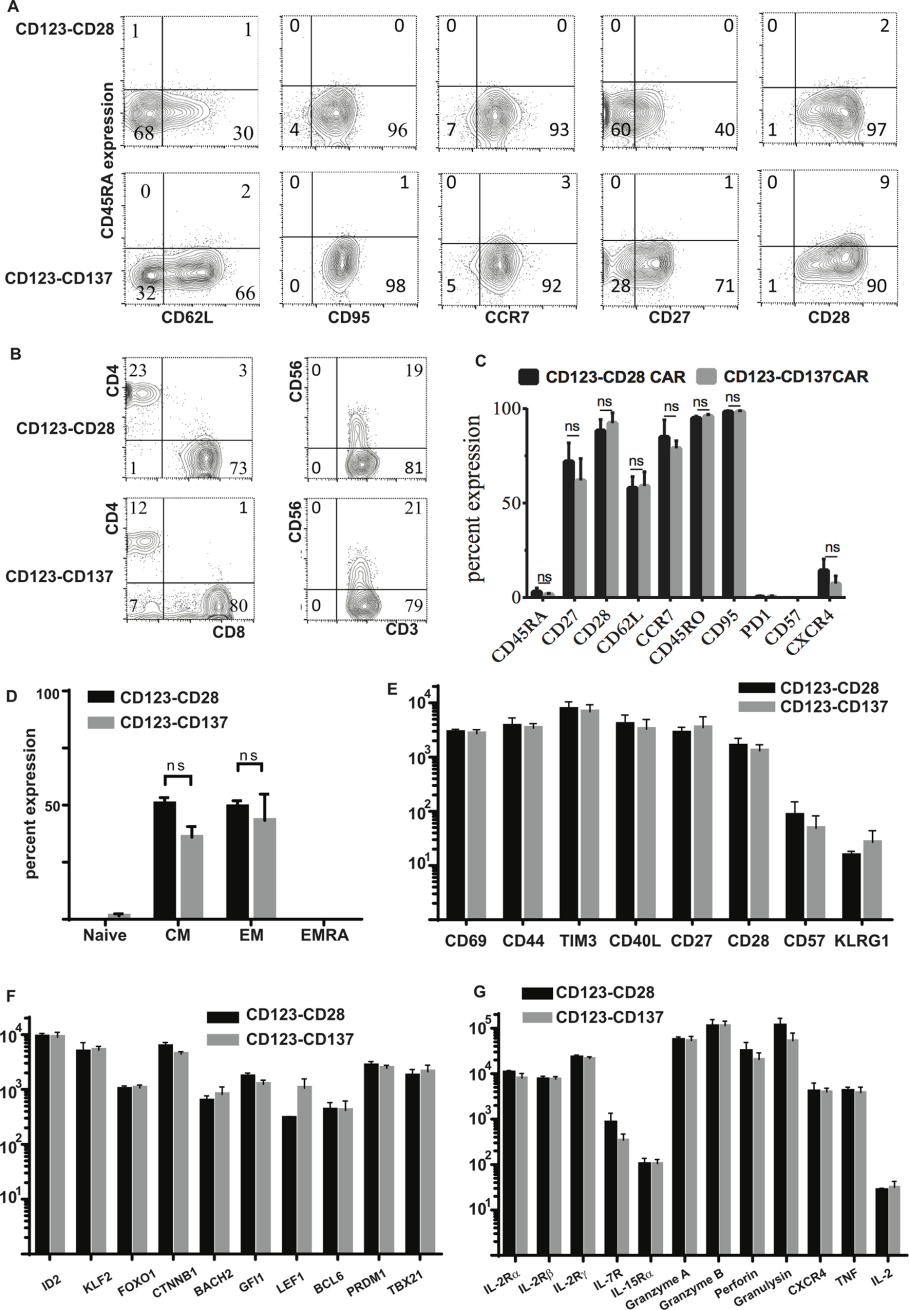
Immunophenotype of CAR^+^ T cells with CD28 or CD137 costimulatory domains. (A) Flow analysis of memory markers on CD3^+^CAR^+^ T cells. Flow cytometry histograms are representative images from one of three donors tested for each memory marker vs. CD45RA (Y axis). (B) Expression of CD4, CD8, and CD56 is shown as in (A) (C) Histograms represent the percentage of CAR^+^ T cells expressing each memory or exhaustion marker (mean ± SEM, n = 3). (D) Histograms represent the percentage of CAR^+^ T cells in each subset, based on flow cytometry phenotype: T_Naïve_ (CD45RA^+^, CD62L^+^, CD95^-^, CCR7^+^), T_EMRA_ (CD45RA^+^, CD62L^neg^, CD95^neg^, CCR7^neg^), T_EM_ (CD45RA^neg^, CD62L^neg^, CD95^+^, CCR7^neg^) and T_CM_ (CD45RA^neg^, CD62L^+^, CD95^+^, CCR7^+^) in CD123-CD28 CAR^+^ T cells (black bars) and CD123-CD137 CAR^+^ T cells (grey bars) (n = 3). Fig 2E, 2F and 2G display the quantitation of mRNA transcripts of lymphocyte genes expressed in CAR T cells as analyzed by non-enzymatic digital multiplex array. (E) Transcriptional profile of activation-, co-stimulation- and exhaustion-related genes. (F) Transcriptional profile of genes associated with differentiation phenotype and memory stage (G) Transcriptional profile of genes for cytokine receptors and markers associated with effector function.

SB transposition and expansion on mIL15^+^AaPC in presence of IL-2 and IL-21 resulted in the outgrowth of T cells expressing markers associated less with a differentiated phenotype and more with an early memory phenotype: CD45RA^lo^, CD62L, CCR7, CD27, and CD28 (**Fig 3A**) with a mixture of CD8^+^ and CD4^+^ T cells **(Fig 3B)**. Most cells expressed CD45RO, but few expressed the bone marrow (BM) homing receptor CXCR4, and none expressed CD57 or PD-1. **(Fig 3C).** Very few CAR^+^ T cells were observed with either a naïve (T_N_) phenotype (CD45RA^+^CD62L^+^CD95^neg^ CCR7^+^) or T_EMRA_ phenotype (CD45RA^+^CD62L^neg^CD95^neg^ CCR7^neg^); the majority were either T_EM_ (CD45RA^neg^CD62L^neg^CD95^+^ CCR7^neg^) or T_CM_ (CD45RA^neg^CD62L^+^CD95^+^ CCR7^+^) (**Fig 3D**) and co-expressed CD27 and CD28 (**Fig 3A**).

The transcriptional profile of CAR^+^ T cells was assessed with a Nanostring digital multiplex mRNA array, which showed expression of the T cell activation markers CD69, CD44, and TIM3, co- stimulatory molecules CD40L, CD27 and CD28, and no expression of the exhaustion and terminal differentiation markers B3GAT1 (Beta-1,3-Glucuronyltransferase-1; CD57) and KLRG*1* **(Fig 3E).** Concurrent expression of transcription factors associated with a less differentiated phenotype, such as ID2 (Inhibitor of DNA Binding-2), KLF2 (Kruppel-like Factor-2), FOXO1 (Forkhead Box- O1), CTNNB*1* (β-Catenin), BACH2 (BTB and *CNC* Homology-2), GFI-*1* (Growth Factor Independence-1), and LEF1 (Lymphoid Enhancer Binding Factor-1), with markers associated with more differentiated memory stages, such as BCL6 (B-cell Lymohoma-6), PRDM1 (BLIMP-1), and TBX21 (T-bet), suggests that the expanded CAR^+^ T cells were a heterogeneous mixture of memory phenotypes **(Fig 3F).** The CAR^+^ T cells also expressed several cytokine receptors, including IL2RA (IL-2-Receptor-α; CD25), IL2RB (IL-2-Receptor-β; CD122), IL2RG (IL-2-Receptor-γ; CD132), IL7R (IL-7-Receptor-α; CD127), and IL15Rα (IL-15-Receptor-α). In addition, the CAR^+^ T cells expressed molecules associated with T cell effector (Granzyme A, Granzyme B, Perforin 1, Granulysin, IFN-γ and TNF*)* functions **(Fig 3G).** In summary, AaPC expanded, IL-2- and IL-21-supplemented CAR^+^ T cells contain sub-populations with desirable phenotypes and gene expression patterns predictive of therapeutic efficacy after adoptive transfer.

### Lysis of AML cells in vitro by CAR^+^ T cells

**Fig 4:**
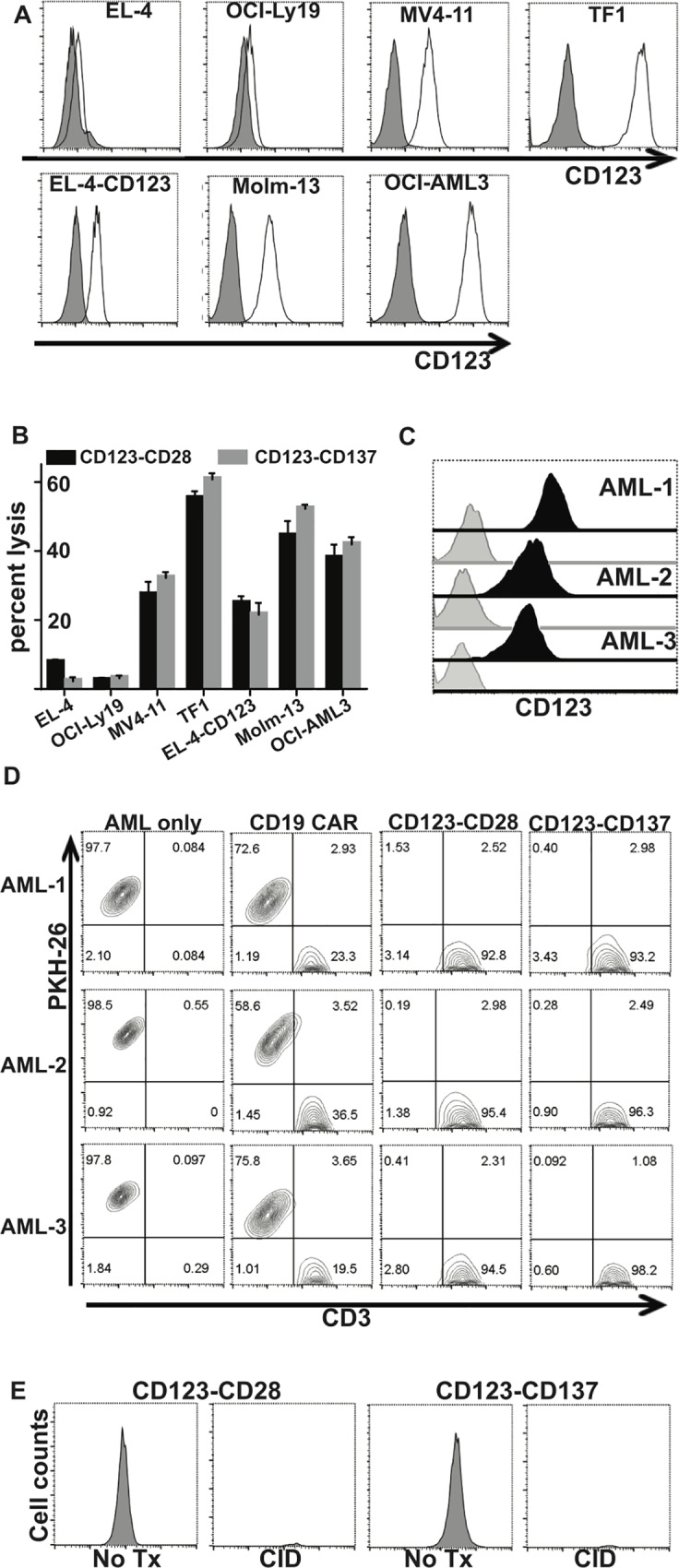
*in vitro* lysis of AML tumor cell lines and primary AML samples. **(A)** Overlay histograms display the flow cytometric analysis of CD123 expression on AML cell lines MV4-11, Molm-13, TF1, OCI-AML3, EL4-Parental and EL4-CD123. Isotype control is shown in grey, and specific staining by the unfilled black line. **(B)** Specific lysis of CD123-CD28 and CD123-CD137 CAR^+^ T cells against AML cell lines EL4, CD123^neg^ OCI-Ly19, MV4-11, TF1, EL4-CD123, Molm-13, and OCI-AML3 assessed with a 4 hour chromium release assay. Histograms represent mean ± SEM, n = 3 **(C)** Flow cytometric analysis of CD123 expression on primary AML samples used in the co-culture assay depicted in **(D)**. Lysis of PKH-26 labeled primary AML cells by CD123-CD28 or CD123-CD137 CAR T cells at 1:1 ratio for 72 hours. CD19-specific CAR^+^ T cells were used as a negative control.

CD123 expression was evaluated on human AML cell lines MV4-11, TF1, Molm-13, OCI-Ly19, and OCI-AML3, and the CD123^neg^ murine T cell lymphoma cell line EL4, as well as EL4 cells transfected with CD123. All cell lines expressed CD123, except parental EL4 cells and OCI-Ly19 **(Fig 4A).** To evaluate the functionality of CD123-specific CAR^+^ T cells *in vitro*, we used a 4-hour chromium release assay for AML cell lines **(Fig 4B)** and a flow cytometry-based killing assay for primary AML cells **(Fig 4C and 4D)**. CD123-specific T cells were able to lyse CD123^+^ AML cell lines but did not kill the CD123^neg^ B-cell lymphoma cell line OCI-Ly19. To provide further evidence that CD123-specific CAR T cells are antigen-specific, we genetically modified EL4 cells to express CD123 (EL4-CD123). CD123-specific T cells efficiently killed EL4-CD123 but not EL4 parental cells. CD123-specific T cells were co-cultured with CD123^+^ primary AML cells for 72 hours, with CD19-specific CAR^+^ T cells used as a negative control, and target cytolysis was assessed by flow cytometry **(Fig 4D)**. CD123-specific CAR^+^ T cells recognized and killed CD123^+^, CD19^neg^ primary AML cells but CD19-specific CAR^+^ T cells did not. The *in vitro* functionality of iCasp9 was assessed by treating CAR^+^ T cells for 24 hours with 1μM AP20187, a synthetic CID, which rapidly eliminated T cells in the CID-treated group **(Fig 4E).** These results indicate that T cells genetically modified to express a CD123-specific CAR kill AML tumor targets in an antigen-specific manner, and can be deleted effectively through iCasp9 suicide switch activation.

### CD123-specific T cells induce AML regression in vivo

**Fig 5:**
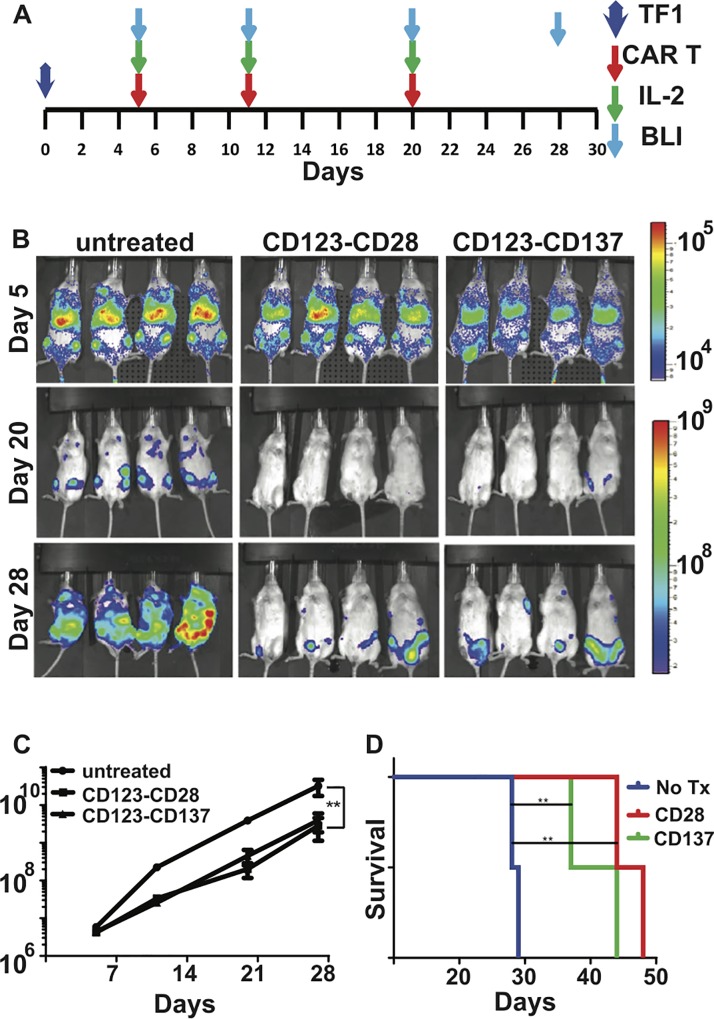
Comparison of costimulatory domains for the treatment of AML using CD123-specific CAR^+^ T cells in a murine model. **(A)** Schematic of the TF1 xenograft model. 2.5 × 10^6^ TF1-*effLuc*-mKate cells were injected intravenously into NSG mice on day 0. On Day 5, tumor engraftment was quantified using non-invasive bioluminescence imaging (BLI), and mice were randomly divided into 3 groups: untreated (control), CD123-CD28-treated, or CD123-CD137-treated. CAR-treated mice were given infusions of T cells followed by IL-2 treatment and BLI on day 5, 11 and 20. Untreated mice received no T cells. **(B)** BLI images of mice display an overlay of luciferase activity, using the color scale shown on the right, displayed over the white-light image of the mice. **(C)** Histograms represent the luciferase activity measured by BLI for each group (** p < 0.01). **(D)** Kaplan-Meier curves display the survival analysis of xenograft mice treated with CD123-specific CAR T cells (** p < 0.01).

A xenograft model of AML was used to test the antitumor activity of CAR^+^ T cells *in vivo*. On day 0, mice were injected with 2.5 × 10^6^ TF1-mKate-effluc cells, which were allowed to engraft for 5 days. On day 5, tumor engraftment was confirmed by BLI, and 10^7^ CD123-CD28 or CD123-CD137 CAR^+^ T cells per mouse were infused together with IL-2 (60,000 units/mouse) **(Fig 5A)**. Untreated mice served as controls. Additional infusions of T cells were given on days 11 and 20, and mice were imaged for tumor burden on days 20 and 28 **(Fig 5B)**. Untreated mice showed continuous tumor growth, as evidenced by increased bioluminescent flux compared with both CAR^+^ T cell-treated groups. Both CD123-CD28 and CD123-CD137 CAR^+^ T cell-treated groups showed similarly reduced tumor burdens compared with the untreated group, as measured by tumor BLI flux **(Fig 5C)**. Treatments with CD123-specific CAR^+^ T cells significantly prolonged survival of mice in both treated groups compared with the control group **(Fig 5D)**. However, the difference in survival between the two groups did not reach statistical significance (p value 0.0598, n = 4 mice per group). Thus a CD123-specific CAR gave an impressive improvement in survival in an experimental AML model regardless of the source of signal 2.

### Efficacy of CD123-CD28 CAR^+^ T cells against B-ALL

**Fig 6:**
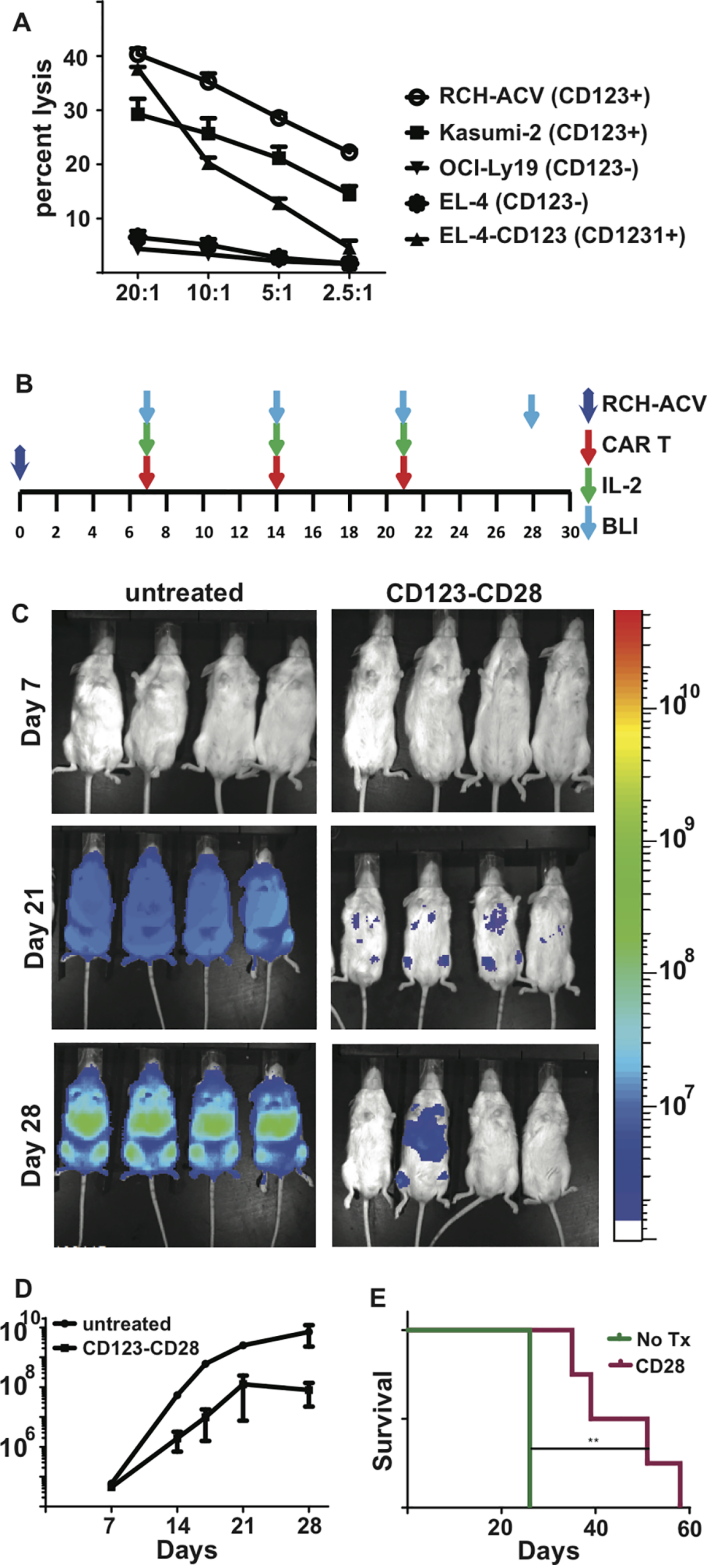
Efficacy of CD123-specific CAR^+^ T cells for the treatment of B-ALL in a murine model. **A)** *In vitro* lysis of B-ALL cell lines by CD123-specific CAR^+^ T cells measured with a 4 hour chromium release assay **(B)** Schematic of the RCH-ACV B-ALL xenograft model. The experimental design is similar to that shown in 5A, but T cells and cytokines were given on days 7, 14 and 21, with imaging weekly. **(C)** BLI imaging of the CAR-treated and untreated groups on day 28. Images are displayed as in 5B. **(D)** Luciferase activity measured by BLI in the CAR-treated group compared with the untreated group. **(E)** Kaplan-Meier curves display the survival analysis of xenograft mice treated with CD123-specific CAR T cells compared with untreated mice. ** p < 0.01.

The data presented thus far demonstrate that a CD123-specific CAR incorporating CD28 as the source of signal 2 was at least equal, and possibly superior, to CARs containing the CD137 costimulatory domain. The eventual clinical utility of CD123-specific CAR T cells would be much greater if these cells also could be used to treat some types of ALL. To assess the utility of CD123 as a target for ALL in our system, we generated CD123-specific CAR T cells with the CD28 co-stimulatory domain, this time without the iCasp9 domain, since it was not needed to address this question. (**S8 Fig.**). CD123 expression by B-ALL cell lines was assessed by flow cytometry **(S9 Fig)** CAR T cells were able to lyse CD123^+^ B-ALL tumor cell lines and CD123-transfected EL4 cells but not parental EL4 cells or CD123^neg^ OCI-Ly19 cells. **(Fig 6A)**. As an *in vivo* model of B-ALL, the cell line RCH-ACV was transduced with effluc **(S7 Fig.)** and engrafted in NSG mice. CD123-specific CAR^+^ T cells were infused one day later, and weekly thereafter for a total of 4 infusions. Control mice were given no T cells **(Fig 6B)**. Tumor burden was measured by BLI on day seven and weekly thereafter **(Fig 6C and 6D)**. Mice given CD123-specific CAR^+^ T cells had a significant improvement in survival **(Fig 6E)** compared with control mice. These data suggest that CD123 may be an alternative TAA for treating B-ALL with CAR^+^ T cells, especially when the B-ALL cells do not express CD19.

## Discussion

One limiting factor in CAR T cell therapy is that TAAs are not specific to tumors, but also may be expressed at low levels on normal cells, potentially resulting in on-target, off-tumor toxicities. Many myeloid antigens, including CD123, previously thought to be restricted to the myeloid lineage, now are known to be expressed on HSCs. Further, CD123-targeted therapies can both eradicate AML tumors and impair normal myelopoiesis [25, 65–67]. Several factors affect the efficacy of CAR T cell treatments, including CAR design, scFv affinity for TAA, and density of TAA on tumor cells. Though the effect of antigen density for CAR therapy is not yet well-defined, it appears that CAR T cells preferably target tumors with high antigen density, while cells with lower density are more resistant to CAR T cells [68, 69]. The affinity of the scFv for TAA also affects the density of TAA required for efficient killing [13]. One potential means of generating scFvs with a range of affinities for TAA is combining the V_H_ and V_L_ chains from different mAbs. Importantly, we show that this approach was able to create functional CARs with efficient target cytolysis. While the efficiency of the resulting CAR^+^ T cells was similar with all CAR designs, this was not surprising, since the original mAbs all resulted in CARs with similar target cytolysis (see Fig 1). By choosing different sources of V_H_ and V_L_ chains and perhaps different hinge regions, we may be able to tune the activation threshold for CAR T cells further, especially if a wider range of antibody affinities is used than was chosen for these studies.

Our constructs also showed specific cytolysis of non-malignant BM cells, both those bearing lineage markers and the lineage-negative cells that presumably represent hematopoietic stem cells. This observation is consistent with the recent report of normal myelopoiesis being severely impaired by CD123-specific CAR therapy in animal models [25]. This TAA-specific, off-tumor effect underscores the need for mechanisms to eliminate CAR-mediated killing once MRD is eliminated. Fortunately, our data show that an inducible suicide switch consisting of an in-frame iCasp9 gene and a Furin/F2A linker did not impair specific cytolysis of CD123-specific CAR^+^ T cells, and that the iCasp9 system was effective in killing CAR^+^ T cells when induced.

CARs that activate through chimeric CD28 or CD137 endo-domains have anti-tumor activity and have facilitated durable remissions in clinical trials, with pros and cons for each design. The optimal source for signal 2 in CAR design currently is unknown, though recent studies demonstrated enhanced persistence for CARs with CD137 compared with those with a CD28 endodomain [12]. Preclinical data that supports targeting CD123 on AML using CARs with either CD28 [70] or CD137 [25] co-stimulatory domains have been reported. When CARs were expressed in T cells using our well-characterized SB gene expression system and expanded and propagated on CD123-expressing AaPC, the resulting T cells were more than 95% CAR^+^ and had a mixture of memory phenotypes, with relatively few naïve or T_EMRA_ cells, and no evidence of exhaustion or terminal differentiation (see Fig 3). The majority of the cells had either a central memory or effector memory phenotype, and there was no significant difference in the phenotype of the resulting T cells based on the presence of CD28 or CD137 signaling domains. In our head- to-head comparison of CD123-specific CARs with CD28 and CD137co-stimulatory domains, we observed similar rates of target lysis with both constructs *in vitro*, though there was a trend toward better survival with the CD28-containing construct in our *in vivo* AML model.

Though CARs typically are identified by their endo-domains and scFv, the other components of CARs, including the hinge/spacer region, also play a crucial role in their function and clinical efficacy. The constant region of IgG4 and CD8α are frequently used extracellular (stalk) hinge regions, though the Fc region has been reported to engage Fc receptors and activate innate immune cells [71]. We showed that CAR constructs using a CD8α-derived hinge provided highly effective cytolysis in our CD123-specific constructs. Interestingly, the choice of spacer had a much greater impact on target cytolysis than expected, with a CAR utilizing a CD8-derived spacer achieving much better *in vitro* cytolysis than the same scFv using an IgG4-derived spacer (CAR6 vs. CAR10, Fig 1D). This observation requires further investigation in future models.

Employing CAR T cells specific for CD123 after hematopoietic transplantation may help eradicate MRD in AML patients, resulting in complete remission. Our data also indicate a possible use in refractory ALL patients. Since some B-ALL patients treated with CD19 CAR^+^ T cells relapsed with CD19^neg^, CD123^+^ tumor cells [11, 45], CD123-specific T cells may provide an alternative for these patients. Certainly the improved survival of mice in the RCH-ACV experimental ALL *in vivo* model suggests that such a use would be beneficial, increasing the pool of patients that might benefit from development of CD123-specific CAR T cell therapy. In summary, our data supports the concept of developing CARs derived from different mAbs specific for the same TAA by combining V_H_ and V_L_ chains, tuning the affinity of the CAR. CARs activated through CD28 or CD137 also showed similar efficacy *in vitro* and *in vivo*, and inclusion of an iCasp9 domain in frame with a Furin/F2A domain did not impair CAR function and generated an effective suicide switch in CAR^+^ T cells. CD123 is a viable antigen for clinical therapy, especially for CAR T cells generated with the SB system, which may have advantages over viral methods of transgene expression.

## Supporting Information

S1 Fig Plasmid maps of *Sleeping beauty* transposons encoding CARs specific for CD123. DNA plasmid vector maps for CD123-CD28 CAR (left) and CD123-CD137 CAR (right). IR/DR: *Sleeping beauty* Inverted Repeats/Direct Repeats, hEF-1alpha/p: human elongation factor-1 alpha promoter, CD123-CD28 CAR: Human codon-optimized CD123-specific CD28 CAR fused to iCasp 9 via a Furin/F2A peptide, CD123-CD137 CAR: Human codon-optimized CD123-specific CD137 CAR fused to iCasp 9 via a Furin/F2A peptide. SIM: “SIM” PCR tracking oligonucleotides, FRA: “FRA” PCR tracking oligonucleotides, BGH polyA: bovine growth hormone polyadenylation sequence, ColE1: A minimal *E*.*coli* origin of replication, Kan/R: Bacterial selection gene encoding kanamycin resistance, Kan/p: Prokaryotic promoter.
(TIFF)

S2 Fig Schematics of mIL15. (A) *Sleeping Beauty* DNA transposon map for mIL15 [IL15-IL15Ra-Flag (CoOp)/pSBSO]. IL-15 is fused with full-length IL-15Rα. hEF-1alpha/p: human elongation factor-1 alpha promoter, TM: transmembrane domain, BGH: polyadenylation signal from bovine growth hormone, IR/DR: *Sleeping beauty* Inverted Repeats/Direct Repeats, ColE1: *E*. *coli* origin of replication, Kan/R: gene encoding kanamycin resistance for bacterial selection, Kan/p: prokaryotic promoter.
(TIF)

S3 Fig DNA plasmid vector map of *Sleeping Beauty* transposon expressing the ROR1 antigen. IR/DR: *Sleeping beauty* Inverted Repeats/Direct Repeats, BGH polyA: Bovine growth hormone polyadenylation sequence, ColE1: A minimal *E*.*coli* origin of replication, Kan/R: Bacterial selection gene encoding kanamycin resistance, Kan/p: Prokaryotic promoter.
(TIFF)

S4 Fig DNA plasmid vector map of *Sleeping Beauty* transposon expressing the CD123 antigen. IR/DR: *Sleeping beauty* Inverted Repeats/Direct Repeats, MNDU3/P: modified myeloproliferative sarcoma virus long terminal repeat enhancer–promoter, CD123: Human codon-optimized CD123 antigen fused to a hygromycin resistance gene through FLAG and a furin/F2A peptide linker. TK: codon-optimized thymidine kinase gene, BGH polyA: Bovine growth hormone polyadenylation sequence, ColE1: A minimal *E*.*coli* origin of replication, Kan/R: Bacterial selection gene encoding kanamycin resistance, Kan/p: Prokaryotic promoter.
(TIFF)

S5 Fig Antibodies used for immunophenotyping of CD123-specific CAR T cells. (DOCX)

S6 Fig Assessment of effluc labeling of TF1 cells for *in vivo* use. The GM-CSF-dependent erythrocytic leukemia cell line TF1 was genetically modified with lentiviral particles to express the mKate fluorescent protein and enhanced firefly luciferase (effluc). Flux intensity was measured using a firefly luciferase assay (**** p < 0.00001 by unpaired t-test).
(TIFF)

S7 Fig Assessment of effluc labeling of RCH-ACV cells for *in vivo* use. Luciferase activity in the B-ALL cell line RCH-ACV transduced with a lentiviral vector expressing firefly luciferase, compared with effluc^neg^ control cells (**** p < 0.00001 by unpaired t-test).
(TIFF)

S8 Fig DNA plasmid vector map of S*leeping Beauty* transposon encoding a CD123-specific CAR with a CD28 co-stimulatory domain, as used in the ALL *in vivo* experiments, Fig 6. This is the same scFv as CAR10 (**Fig 1**). IR/DR: *Sleeping Beauty* Inverted Repeat/Direct repeats, ColE1: A minimal *E*.*coli* origin of replication, Kan/R: Bacterial selection gene encoding kanamycin resistance, Kan/p: Prokaryotic promoter. hEF-1alpha/p: human Elongation Factor-1 alpha region promoter
(TIFF)

S9 Fig Flow cytometry analysis of CD123 expression on B-ALL cell lines RCH-ACV, KASUMI-2, and Nalm6, on the B-cell lymphoma cell lines OCI-Ly19 and EL4 (parental and CD123-expressing). (TIFF)
