# Supplementary figures and images for: Redirecting Specificity of T cells Using the Sleeping Beauty System to Express Chimeric Antigen Receptors by Mix-and-Matching of VL and VH Domains Targeting CD123+ Tumors
Source: PLoS One. 2016 Aug 22;11(8):e0159477. doi: 10.1371/journal.pone.0159477 (PMC4993583; doi:10.1371/journal.pone.0159477)

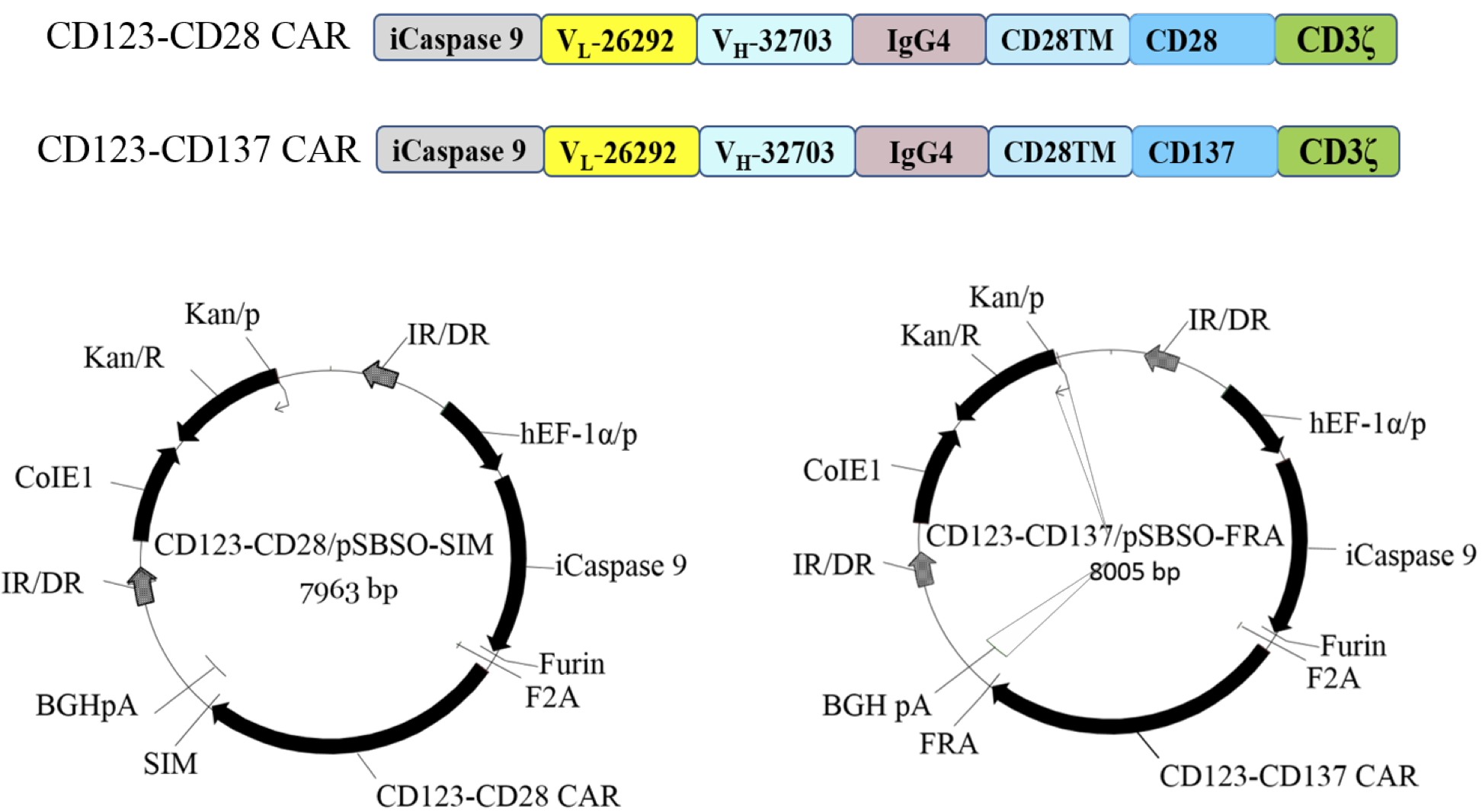

Supplement: S1 Fig — DNA plasmid vector maps for CD123-CD28 CAR (left) and CD123-CD137 CAR (right). IR/DR: Sleeping beauty Inverted Repeats/Direct Repeats, hEF-1alpha/p: human elongation factor-1 alpha promoter, CD123-CD28 CAR: Human codon-optimized CD123-specific CD28 CAR fused to iCasp 9 via a Furin/F2A peptide, CD123-CD137 CAR: Human codon-optimized CD123-specific CD137 CAR fused to iCasp 9 via a Furin/F2A peptide. SIM: “SIM” PCR tracking oligonucleotides, FRA: “FRA” PCR tracking oligonucleotides, BGH polyA: bovine growth hormone polyadenylation sequence, ColE1: A minimal E.coli origin of replication, Kan/R: Bacterial selection gene encoding kanamycin resistance, Kan/p: Prokaryotic promoter. (TIFF) [file pone.0159477.s001.tiff]

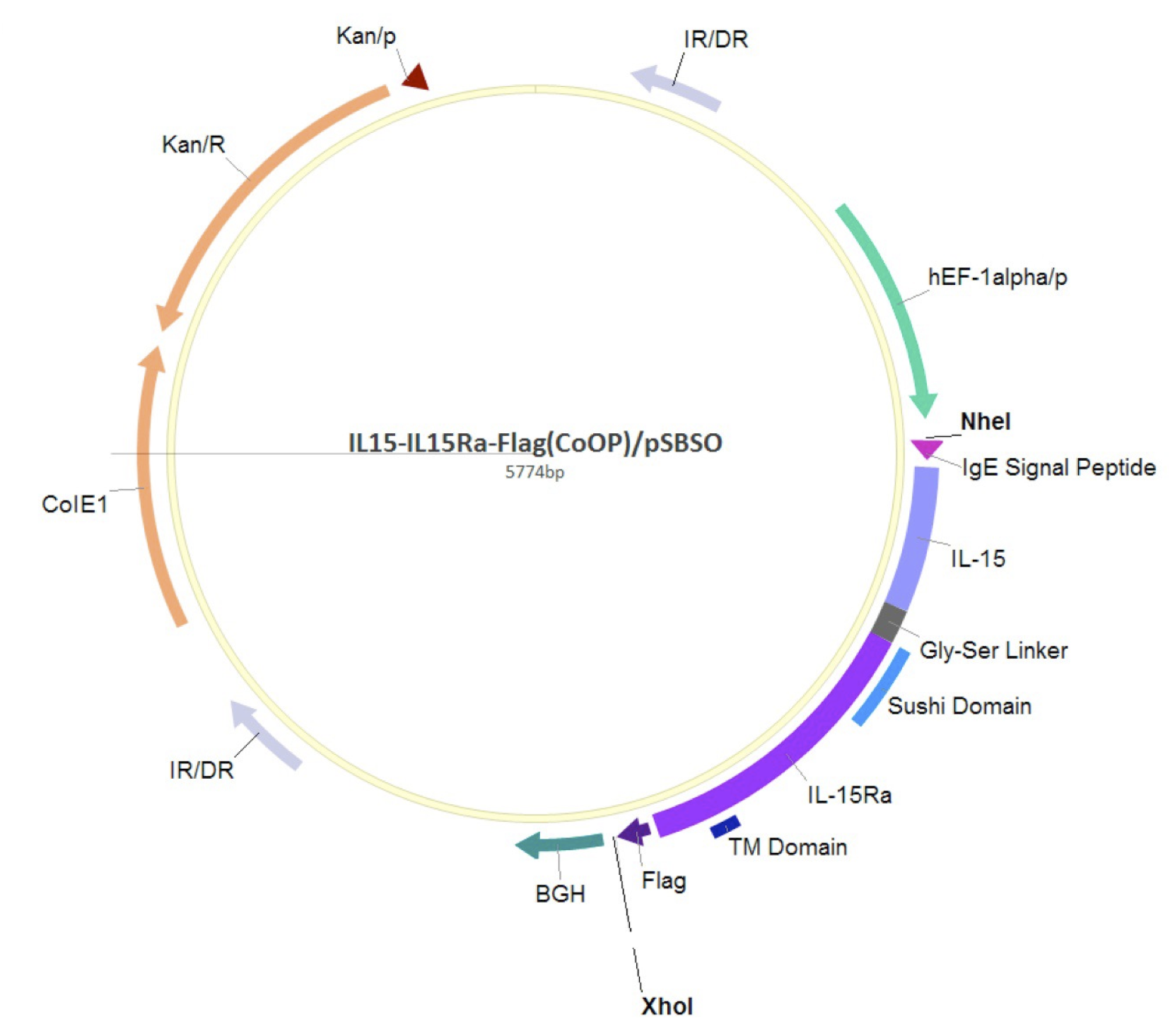

Supplement: S2 Fig — (A) Sleeping Beauty DNA transposon map for mIL15 [IL15-IL15Ra-Flag (CoOp)/pSBSO]. IL-15 is fused with full-length IL-15Rα. hEF-1alpha/p: human elongation factor-1 alpha promoter, TM: transmembrane domain, BGH: polyadenylation signal from bovine growth hormone, IR/DR: Sleeping beauty Inverted Repeats/Direct Repeats, ColE1: E. coli origin of replication, Kan/R: gene encoding kanamycin resistance for bacterial selection, Kan/p: prokaryotic promoter. (TIF) [file pone.0159477.s002.Tif]

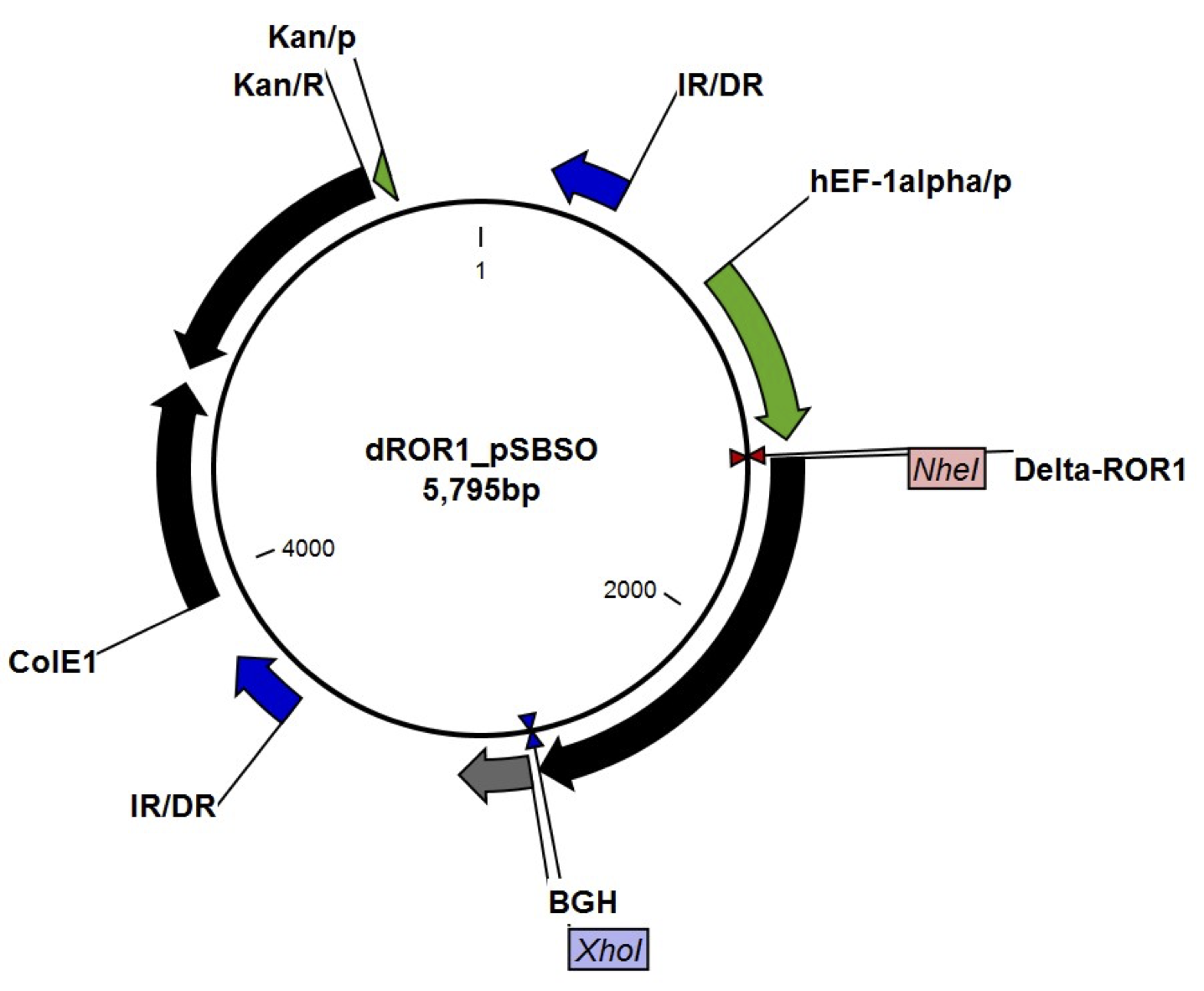

Supplement: S3 Fig — IR/DR: Sleeping beauty Inverted Repeats/Direct Repeats, BGH polyA: Bovine growth hormone polyadenylation sequence, ColE1: A minimal E.coli origin of replication, Kan/R: Bacterial selection gene encoding kanamycin resistance, Kan/p: Prokaryotic promoter. (TIFF) [file pone.0159477.s003.tiff]

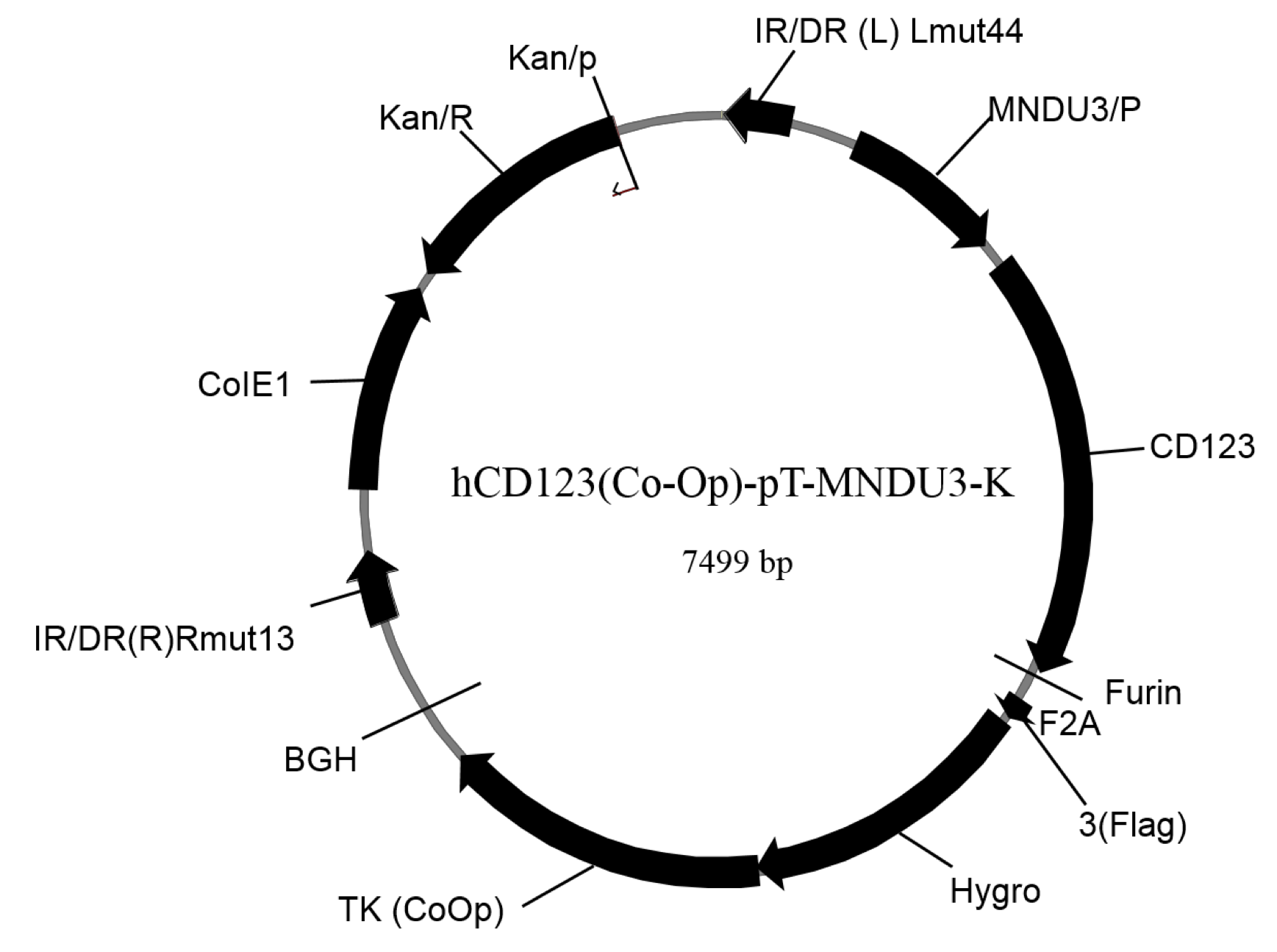

Supplement: S4 Fig — IR/DR: Sleeping beauty Inverted Repeats/Direct Repeats, MNDU3/P: modified myeloproliferative sarcoma virus long terminal repeat enhancer–promoter, CD123: Human codon-optimized CD123 antigen fused to a hygromycin resistance gene through FLAG and a furin/F2A peptide linker. TK: codon-optimized thymidine kinase gene, BGH polyA: Bovine growth hormone polyadenylation sequence, ColE1: A minimal E.coli origin of replication, Kan/R: Bacterial selection gene encoding kanamycin resistance, Kan/p: Prokaryotic promoter. (TIFF) [file pone.0159477.s004.tiff]

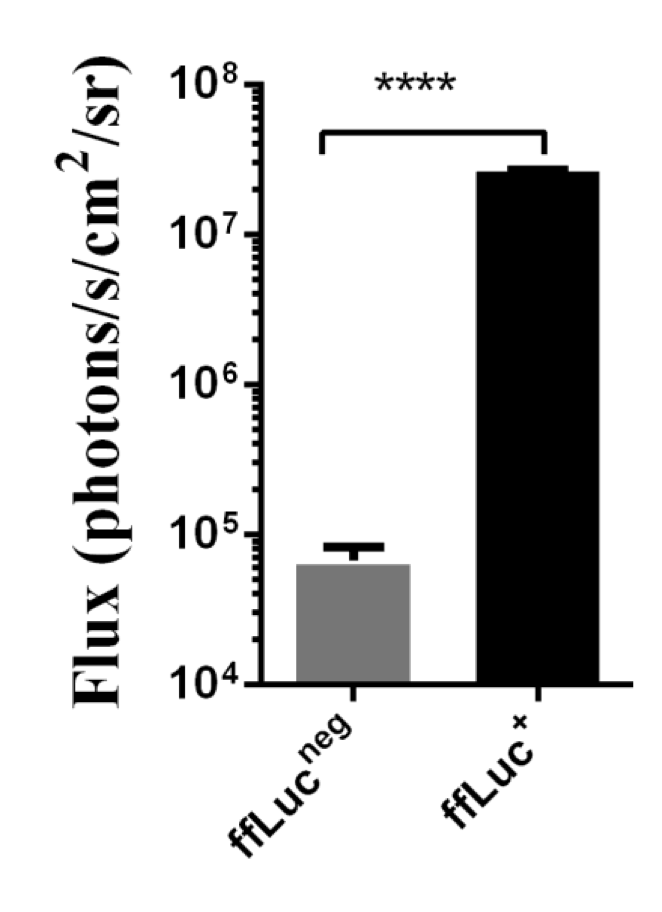

Supplement: S6 Fig — The GM-CSF-dependent erythrocytic leukemia cell line TF1 was genetically modified with lentiviral particles to express the mKate fluorescent protein and enhanced firefly luciferase (effluc). Flux intensity was measured using a firefly luciferase assay (**** p < 0.00001 by unpaired t-test). (TIFF) [file pone.0159477.s006.tiff]

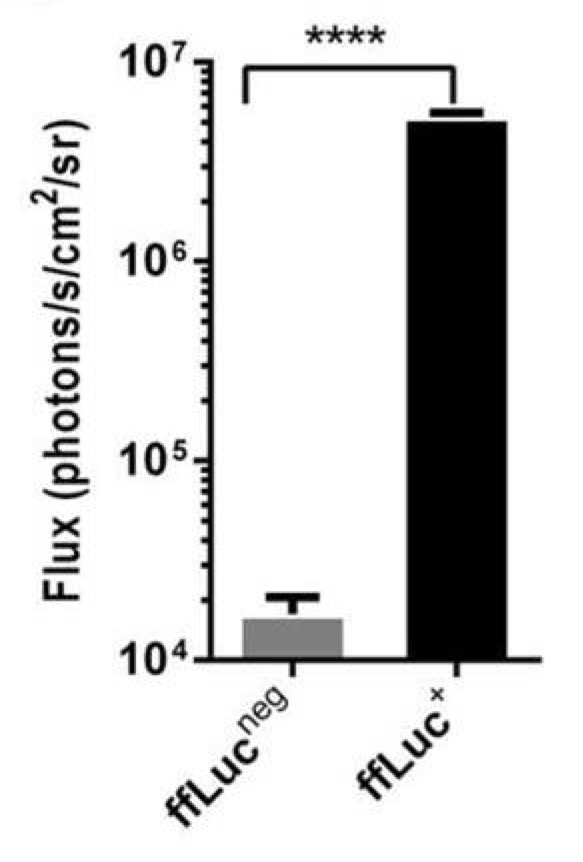

Supplement: S7 Fig — Luciferase activity in the B-ALL cell line RCH-ACV transduced with a lentiviral vector expressing firefly luciferase, compared with efflucneg control cells (**** p < 0.00001 by unpaired t-test). (TIFF) [file pone.0159477.s007.tiff]

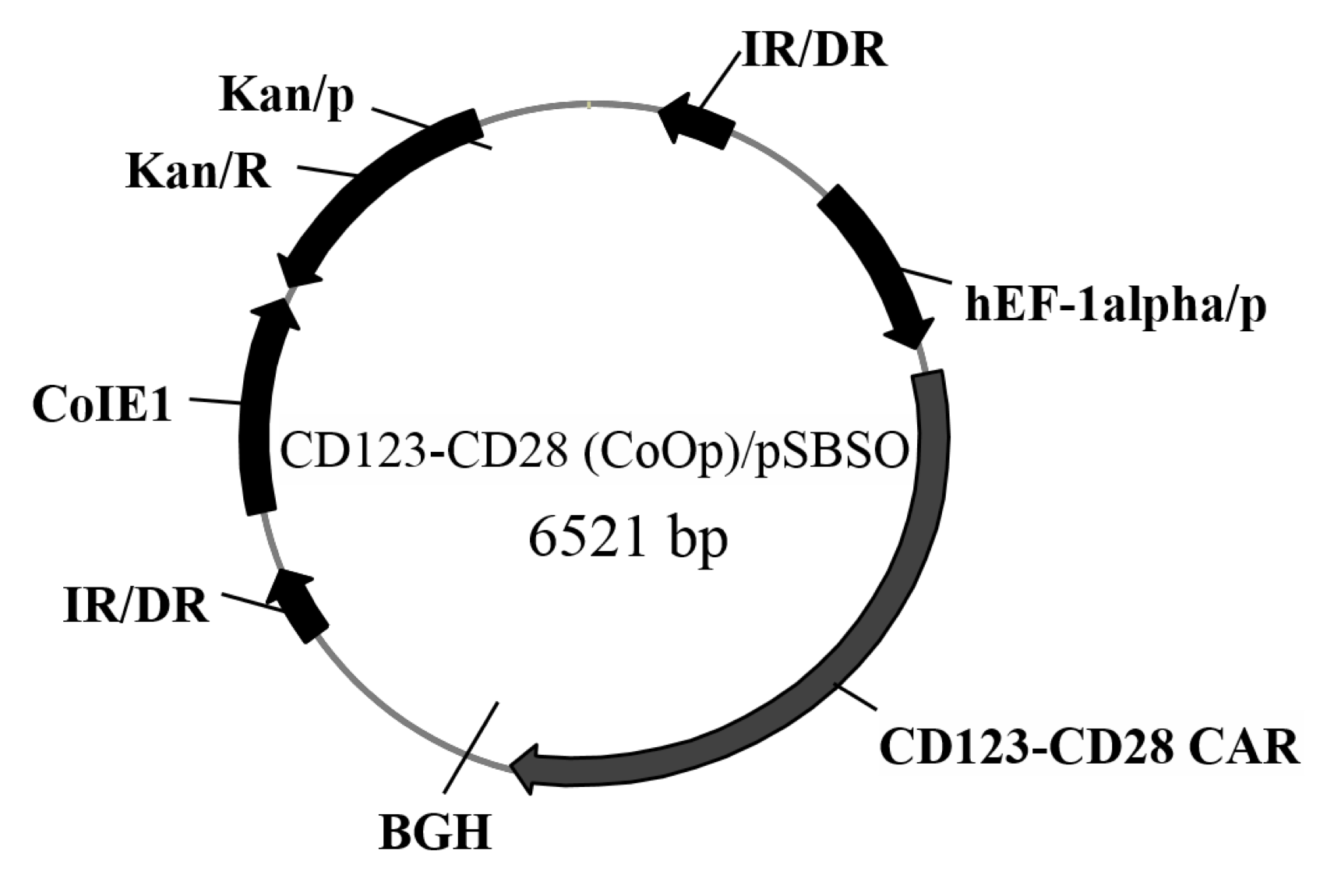

Supplement: S8 Fig — This is the same scFv as CAR10 (Fig 1). IR/DR: Sleeping Beauty Inverted Repeat/Direct repeats, ColE1: A minimal E.coli origin of replication, Kan/R: Bacterial selection gene encoding kanamycin resistance, Kan/p: Prokaryotic promoter. hEF-1alpha/p: human Elongation Factor-1 alpha region promoter (TIFF) [file pone.0159477.s008.tiff]

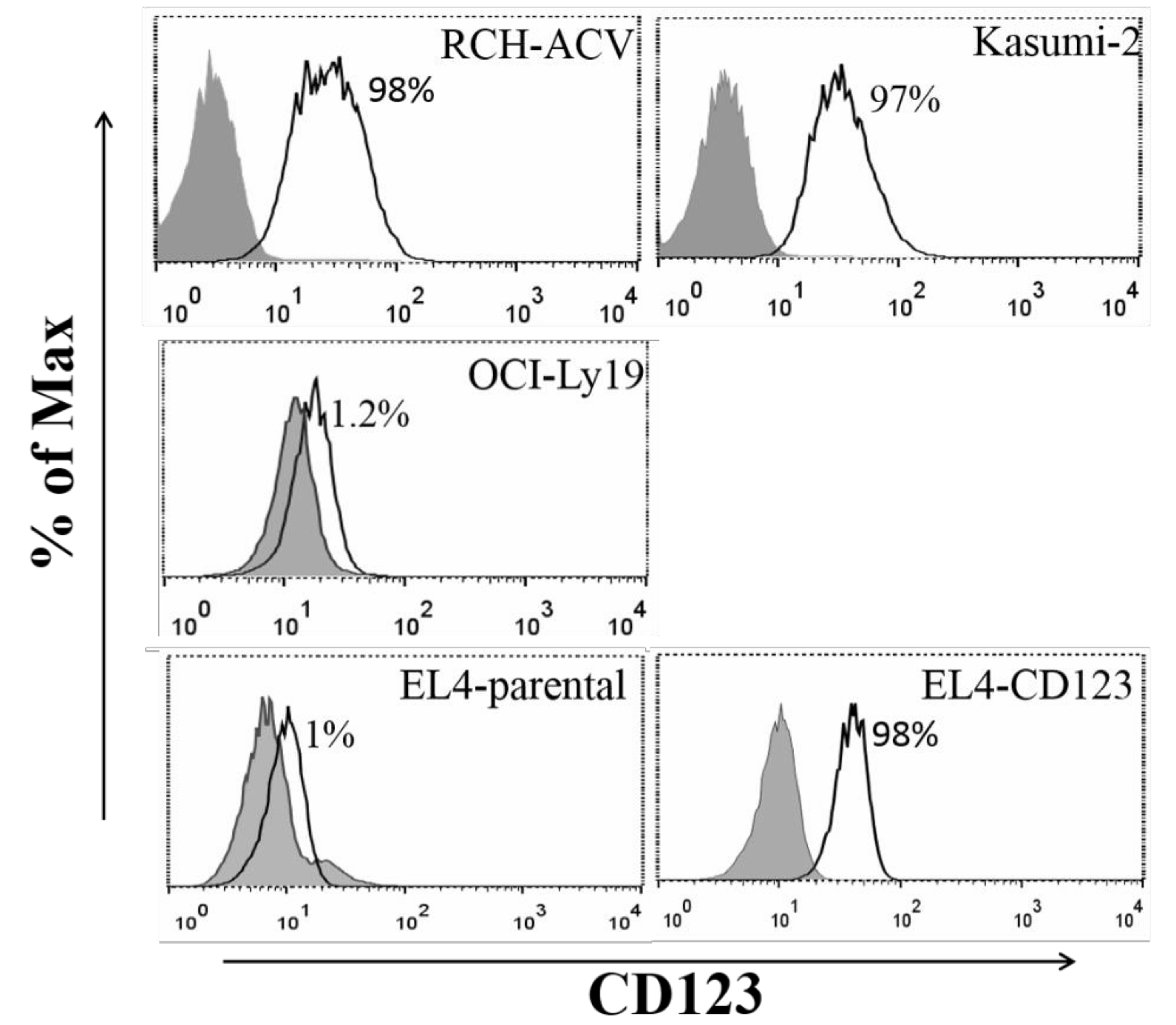

Supplement: S9 Fig — (TIFF) [file pone.0159477.s009.tiff]
