# Supplementary material for: Redirecting Specificity of T cells Using the Sleeping Beauty System to Express Chimeric Antigen Receptors by Mix-and-Matching of VL and VH Domains Targeting CD123+ Tumors
Source: PLoS One. 2016 Aug 22;11(8):e0159477. doi: 10.1371/journal.pone.0159477 (PMC4993583; doi:10.1371/journal.pone.0159477)
Supplement: S5 Fig — (DOCX) [file pone.0159477.s005.docx]

Figure S5

Supplemental Table 1.

**Antibodies used for immunophenotyping of CD123-specific CAR T cells**

| **Antibody** | **Catalog #** | **Source** |
| --- | --- | --- |
| CD123 Recombinant fused to Fc | 10518-H03H-20 | Sino biologicals |
| CD3 | 552852 | BD Biosciences |
| Fc | H10104 | Invitrogen |
| CD56 | 340410 | BD Biosciences |
| CD4 | 341654 | BD Biosciences |
| CD8 | 340659 | BD Biosciences |
| CD45RA | 555488 | BD Biosciences |
| CD27 | 558664 | BD Biosciences |
| CD28 | 555729 | BD Biosciences |
| CD45RO | 555492 | BD Biosciences |
| CD62L | 555544 | BD Biosciences |
| CCR7 | 335605 | Biolegend |
| CD95 | 558814 | BD Biosciences |
| CD57 | 555619 | BD Biosciences |
| PD1 | 557860 | BD Biosciences |
| CXCR4 | 555974 | BD Biosciences |
